# Robust Cell Segmentation for Size Distribution Estimation via Synthetic‐Data Training

**DOI:** 10.1002/biot.70272

**Published:** 2026-07-09

**Authors:** Han Bit Kim, Chaeeun Lee, Naeun Lee, Hyeongseok Han, Chanhun Park, Moo Sun Hong

**Affiliations:** ^1^ Department of Chemical and Biological Engineering Seoul National University Seoul Republic of Korea; ^2^ CJ BIO Research Institute CJ CheilJedang Suwon‐Si Gyeonggi‐do Republic of Korea

**Keywords:** annotation‐free, cell segmentation, online monitoring, PHA, synthetic data

## Abstract

Polyhydroxyalkanoates (PHAs) are biodegradable and biocompatible plastics, yet large‐scale production remains limited by costly batch operations and the lack of online analytical tools. Monitoring cell size distribution serves as a strong surrogate for intracellular PHA content, but training robust cell segmentation models for industrial bioprocesses requires extensive manual annotation, which is highly labor‐intensive and impractical for densely populated microscopy images. To address this critical bottleneck, an annotation‐free cell segmentation and size estimation pipeline tailored for real time monitoring is presented. Rather than relying on architectural modifications, this framework introduces a system‐level automated training strategy: individual cells are automatically extracted from diluted‐sample microscopy images using edge enhancement and rule‐based binarization, then augmented and composited onto heterogeneous backgrounds to emulate the texture of undiluted, dense cultures. This fully synthetic data generation eliminates the need for manual labeling while enabling robust training of instance segmentation models. When implemented using a Mask R‐CNN backbone, the proposed pipeline consistently tracks flow cytometry forward‐scatter (FSC) distribution trends under diverse imaging conditions and achieves higher correlation with FSC data compared to Cellpose and CellSAM, representative foundation models for cell segmentation. This annotation‐free methodology provides a practical and reliable automated online monitoring solution for intelligent PHA manufacturing.

## Graphical Abstract and Lay Summary

This study presents an annotation‐free cell segmentation pipeline for the real‐time monitoring of microbial cell size distributions. By automatically extracting single cells from diluted samples using edge detection and compositing them to create dense synthetic training data, a Mask R‐CNN model was trained without manual labeling. The proposed method successfully handled diverse image distribution shifts, accurately tracking cell size trends and demonstrating greater robustness than zero‐shot foundation models.

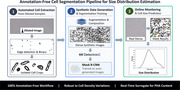

## 1 Introduction

Polyhydroxyalkanoates (PHAs) are renewable, biodegradable, and biocompatible polymers produced by microorganisms and are increasingly recognized as sustainable alternatives to conventional petrochemical plastics. Despite their potential, large‐scale production remains limited by high costs associated with substrate purity, batch‐based cultivation, and downstream processing complexity. Overcoming these limitations requires reliable real time monitoring, optimization, and quality control strategies to improve productivity and cost efficiency for industrial implementation [1, 2].

Industrial PHA production still relies predominantly on offline analytical assays. Gas and liquid chromatography are widely used for quantitative determination of PHA content, while nuclear magnetic resonance (NMR) and mass spectrometry are employed to characterize polymer composition and molecular structure. However, these conventional techniques require depolymerization of the sample and are therefore unsuitable for in‐line monitoring. The development of online analytical methods is essential to enable real time process control and ultimately achieve intelligent, fully automated PHA manufacturing [3]. Image‐based analysis of cell size distribution offers a promising approach for such online measurement, as several studies have reported that microorganisms tend to increase in size during PHA accumulation, indicating a positive correlation between cell size and intracellular PHA content [4]. While flow cytometry is a reliable commercial tool for measuring cell size, it is inherently an offline analytical method that requires manual sampling and extensive dilution, limiting its suitability for continuous, real time applications. In contrast, image‐based analysis enables in situ integration, supporting automated monitoring and real time feedback control without additional sample preparation.

Various cell segmentation models with strong generalizability, such as Cellpose [5], CellSAM [6], Omnipose [7], and the Segment Anything Model (SAM) [8], have been developed to enable accurate segmentation across diverse cell types without additional training. However, due to the substantial variability in real microscopy images obtained from industrial PHA production environments, these models often exhibit inconsistent performance and sensitivity to image distribution shifts under varying imaging conditions, thereby reducing segmentation accuracy for specific tasks. Such distributional differences can arise not only from background texture and illumination but also from variations in cell density. For instance, as demonstrated in subsequent evaluations, state‐of‐the‐art (SOTA) models like CellSAM and Cellpose often over‐segment cell instances in sparsely populated images. The results indicate that model performance can vary significantly depending on image characteristics such as density, highlighting their limited domain generalization. Consequently, task‐specific fine‐tuning is required to achieve reliable performance, which in turn involves the construction of extensive datasets for effective training [9].

Datasets for training instance segmentation models are typically constructed through either manual annotation or synthetic image generation using simulation tools. However, manual annotation is both time‐consuming and labor‐intensive, becoming particularly impractical when object boundaries are ambiguous or images are densely populated with overlapping structures. Moreover, as new data continuously emerge, maintaining large annotated datasets becomes increasingly infeasible. To address these challenges, numerous studies have explored synthetic data generation as an alternative, emphasizing the importance of creating images that closely resemble real microscopy data. To this end, physics‐based simulators [10, 11, 12] and deep learning‐based generative models [13, 14, 15, 16] have been employed to construct training datasets, resulting in substantial improvements in segmentation accuracy. Nonetheless, synthetic images generated through simulation frequently differ from real microscopy data in terms of texture and distribution, and deep learning‐based generative approaches often rely on annotated data or prior supervision, which limits their practicality for continuous and scalable dataset construction.

Therefore, the core contribution of this work lies in the development of a practical, annotation‐free pipeline for generating training data and monitoring cell sizes in real time. To overcome the impossibility of manually labeling dense images, the proposed framework leverages sparsely populated (diluted) samples. By applying continuous edge enhancement and rule‐based binarization, precise single‐cell crops can be automatically extracted. These cells are then composited onto heterogeneous backgrounds to reconstruct realistic dense‐image conditions. This fully automated synthetic data pipeline eliminates the manual labeling barrier, enabling robust training of instance segmentation models for continuous PHA process feedback. Building on this framework, a robust cell segmentation pipeline trained on synthetic data is presented, demonstrating accurate estimation of cell size distributions and their use for PHA content, enabling reliable online monitoring and real time process feedback.

## 2 Materials and Methods

### 2.1 Materials and Experimental Methods

A recombinant *Escherichia coli (E. coli)* strain was cultivated for PHA production across multiple batches using different 2000 L and 3000 L industrial‐scale bioreactors, as well as one 5 L laboratory‐scale bioreactor used for synthetic data generation. Specific genetic modifications of the strain are proprietary and therefore not disclosed. Fermentation conditions, including sugar types, media configurations, and oxygen transfer rates, were adjusted across batches, resulting in variations in broth color and background turbidity that provided a diverse set of imaging conditions. Samples were collected at equal time intervals and labeled chronologically as S1‐S11, with S1 denoting the earliest sampling point. Intracellular PHA content was quantitatively analyzed using gas chromatography (GC) (GC‐2010 Pro, SHIMADZU, Japan) with flame ionization detection (FID) after freeze‐drying and butanolysis of the cells.

For synthetic‐image generation, 100‐fold and 250‐fold serial dilutions were prepared. The 100‐fold dilution was obtained by two consecutive 10‐fold dilutions of 1 mL of culture broth. The 250‐fold dilution was prepared by two 10‐fold dilutions followed by mixing 400 µL of the resulting suspension with 600 µL of phosphate‐buffered saline (PBS) to achieve an additional 2.5‐fold step. Six independent batches were used to prepare 100‐fold dilutions, and three of these were also used to prepare 250‐fold dilutions. Batches used solely for synthetic‐image generation were excluded from those reported in the main results.

Microscopy images were acquired using an Olympus BX53 optical microscope at 100 × magnification with an oil immersion objective, with a spatial calibration of 0.09 µm pixel^−1^. For image acquisition, 4 µL of the sample was placed on a glass slide, covered with a coverslip, and gently pressed to form a thin layer. The focus was continuously adjusted during imaging to compensate for sample drift. A total of 2913 images were collected across all batches and sampling time points by acquiring multiple non‐overlapping fields of view for each sample. Forward scatter (FSC) intensities were measured using a BD Accuri C6 Plus personal flow cytometer after 5000‐fold dilution and used as a proxy for relative cell size in correlation analyses.

### 2.2 Computational Methods

**FIGURE 1:**
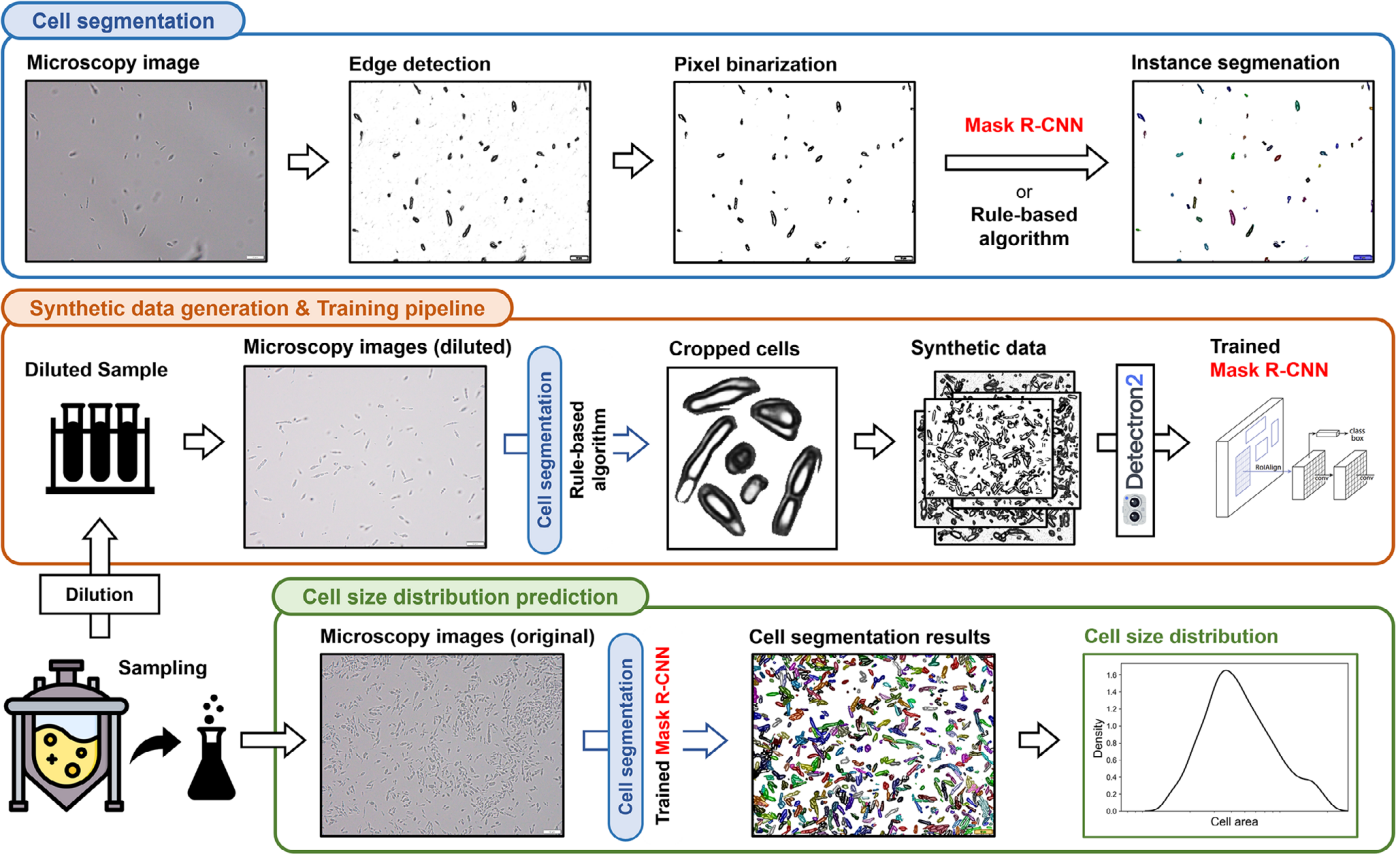
Overall workflow of the proposed system for predicting cell size distribution from microscopy images.

The overall computational workflow is illustrated in Figure 1, which comprises three main steps: cell segmentation, training pipeline, and prediction of the cell size distribution.

For cell segmentation, a robust and fully automated pipeline was developed that integrates edge detection, pixel binarization, and instance segmentation. The edge enhancement and binarization modules emphasize cellular morphology while suppressing low‐frequency background signals. In the training stage, to enable instance segmentation model training on realistic data without manual annotation, a synthetic data generation process was devised based on real microscopy images rather than pure simulation. Samples collected from the fermentor are diluted, and the microscopy images of these diluted samples are processed using a rule‐based segmentation approach to automatically extract reliable single‐cell crops. These cropped cells are then geometrically and photometrically augmented and composited onto heterogeneous backgrounds to emulate undiluted samples with realistic image texture. The Mask R‐CNN architecture is trained on the resulting synthetic image‐mask pairs to improve robustness under varying imaging conditions.

Subsequently, the trained model is applied to real microscopy images, and cell size distributions are estimated from the segmented cell areas. Inferred cell size distributions are visualized using kernel density estimation (KDE) on a log‐scaled axis to obtain smooth and comparable curves, serving as a microscopy‐based surrogate for cell size. For comparison and validation, the forward scatter (FSC) distribution measured by flow cytometry is also reported as an experimental surrogate of the underlying true cell size distribution. After removing low‐intensity noise and smoothing the log‐transformed FSC histogram using KDE, a cleaner reference distribution is obtained. Details of each step are described below.

#### 2.2.1 Cell Segmentation

The cell segmentation process consists of edge detection, pixel binarization, and instance segmentation. Edge detection is performed using the Dense Extreme Inception Network (DexiNed), which preserves continuous boundaries while suppressing noise in challenging microscopy images [17]. This step converts RGB images to grayscale and removes background artifacts. A pretrained DexiNed model available on GitHub^1^ is used for this purpose. The resulting edge map is then binarized with a threshold value of 100 to retain edge features while eliminating residual noise. The binarized edge image serves as the input for instance segmentation.

For instance segmentation, two distinct approaches are employed at different stages of the pipeline. A rule‐based algorithm is used to extract isolated single cells from diluted samples for synthetic data generation, whereas the Mask R‐CNN model is trained on this dataset and applied to segment cells in un‐diluted microscopy images. The rule‐based approach is implemented using OpenCV (open source computer vision library) [18]. From the binarized image, external contours are extracted using cv2.findContours. For each contour *c*, its convex hull *h* and areas *A_c_* =  area(*c*) and *A_h_* =  area(*h*) are computed. Contours with *A_c_*/*A_h_* < 0.70 are excluded to remove irregular or merged shapes, while those with *A_c_* ≤ 50 pixels or *A_c_* ≥ 3000 pixels are filtered out to eliminate spurious small objects and oversized artifacts. Because cell overlap is minimal in diluted‐sample images, this rule‐based method provides segmentations close to the true instances. Accordingly, rule‐based segmentation is used to generate synthetic training data for Mask R‐CNN, while the trained Mask R‐CNN model is used to predict cell size distributions from real microscopy images.

Mask R‐CNN is well suited for this application because it accurately detects and masks individual objects, even when they overlap [19]. The integration of edge‐based preprocessing with instance segmentation has been shown to improve boundary extraction in noisy microscopic environments, such as in protein crystallization analysis [11]. This architecture aligns naturally with the proposed preprocessing steps (edge detection and binarization), enabling robust segmentation under diverse image conditions. In contrast, general‐purpose models such as Cellpose and CellSAM are designed for end‐to‐end inference on raw microscopy images and are less compatible with such preprocessing. Cellpose predicts directly from unprocessed images in a single step, as the model infers spatial gradients and incorporates its own denoising network [5]. Preprocessing operations that remove gradient information can therefore impair its accuracy. Similarly, CellSAM relies on a foundational vision model that requires rich texture and intensity features to compute accurate image embeddings [6], which may be degraded by edge‐based preprocessing. For this reason, Mask R‐CNN is adopted and fine‐tuned as the instance segmentation model, while Cellpose and CellSAM are included as representative SOTA benchmarks.

To further assess the impact of task‐specific training under the proposed preprocessing scheme, the Cellpose model was also fine‐tuned on the same synthetic datasets generated by the pipeline. This comparison evaluates whether fine‐tuning can mitigate the limitations of gradient‐dependent models when applied to edge‐detected and binarized inputs.

Although Mask R‐CNN is employed in this study, the proposed preprocessing and training pipeline is not restricted to a specific architecture and can be extended to other trainable instance segmentation models.

#### 2.2.2 Synthetic Data Generation and Model Training Pipeline

The synthetic training dataset is generated from microscopy images of diluted samples. Cell segmentation using the rule‐based algorithm was applied to 2913 sparse images, yielding 79,196 cropped cells. From these crops, 50–500 cells are randomly selected per synthetic image and augmented using Albumentations [20] and OpenCV to increase data variability and reduce overfitting. Augmentation includes operations such as HorizontalFlip, VerticalFlip, Rotate, GridDistortion, ElasticTransform, GaussianBlur and RandomBrightnessContrast from Albumentations, as well as sharpening, binarization, and resizing from OpenCV.

The augmented cells are composited onto a white canvas with width and height randomly sampled between 300 and 800 pixels. Because Detectron2 dynamically resizes input images during training such that the shorter edge is scaled to 800 pixels, this process intentionally induces image quality variations and shape augmentations. This intrinsic augmentation is designed to help the model better accommodate realistic optical and morphological fluctuations in actual microscopy images. A placement is rejected if the overlap with any existing cell exceeds 20% of either object's area. The composite image is further augmented with Gaussian noise, brightness and contrast adjustment, and salt‐and‐pepper noise to improve robustness and emulate the appearance of undiluted microscopy images. In total, 5000 synthetic images were generated to train the Mask R‐CNN model.

During dataset generation, annotation masks are automatically produced because the cell boundaries are known from the compositing process. The Mask R‐CNN model is trained using Detectron2, an open‐source framework developed by Facebook AI Research (FAIR)^2^. The architecture employs a ResNet‐101 backbone [21] initialized with weights pre‐trained on the MS‐COCO dataset [22].

#### 2.2.3 Cell Size Distribution Prediction

FSC intensities often exhibit a bimodal distribution in which a low‐intensity peak corresponds to background noise or debris and a higher‐intensity peak represents true cellular signals. To suppress the low‐intensity component without imposing a hard cutoff, a probabilistic resampling approach based on a two‐component Gaussian mixture model (GMM) is applied.

Given one‐dimensional FSC data xεR, nonfinite values are removed and a monotonic logarithmic transform z=log(1+x+δ) is applied, where δ ≥ 0 ensures a positive argument. A two‐component GMM is fitted, and the components are ordered such that μ_1_ < μ_2_; the lower‐mean component represents noise, and the higher‐mean component represents cellular signals. The posterior probability of belonging to the signal component is computed as:

$$P\left(\mathrm{signal}\;|\;z\right)=\frac{\pi_{2}N\left(z;\mu_{2},\sigma_{2}^{2}\right)}{\pi_{1}N\left(z;\mu_{1},\sigma_{1}^{2}\right)+\pi_{2}N\left(z;\mu_{2},\sigma_{2}^{2}\right)}$$

A soft weight function is defined as:

$$w\left(x\right)={\left[P\left(\mathrm{signal}\;|\;z\right)\right]}^{1/T},$$

where *T* > 0 controls the transition sharpness; larger *T* values yield smoother, more conservative attenuation. A value of *T*  =  1.5 is used, selected empirically to suppress low‐intensity noise while minimizing data loss. Each value *x* is independently retained with probability *w*(*x*) and otherwise discarded.

Cell size distributions are predicted directly from microscopy images. Samples are collected from the reactor, and multiple images are obtained for each sample. Cell segmentation using the trained Mask R‐CNN model is applied to each image, and segmented cell areas are aggregated across all fields of view. The histogram of segmented cell areas represents the estimated cell size distribution. To visualize and compare these distributions, KDE is employed as a nonparametric smoothing method that produces continuous curves by averaging localized kernels centered at each observation, as shown in Figure 3.

## 3 Results

### 3.1 Qualitative Assessment of Segmentation and Distribution Estimation

**FIGURE 2:**
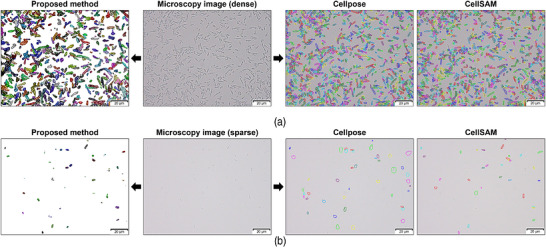
Comparison of cell segmentation results on (a) densely and (b) sparsely populated microscopy images. The left images (proposed method) display segments filled with distinct colors, whereas the right images (Cellpose and CellSAM) present segments outlined in unique colors.

Figure 2 presents representative cell segmentation results obtained using the proposed method and two foundation models (Cellpose and CellSAM) across both densely and sparsely populated microscopy images. For dense images, all three models perform qualitatively well overall; however, the proposed method occasionally merges overlapping cells into a single object, whereas the foundation models tend to over‐segment individual cells into multiple fragments. In contrast, for sparse images, the proposed model continues to produce plausible segmentations, while the foundation models yield masks substantially larger than the true objects. These behaviors are consistently observed in images containing smaller cells, indicating that the performance of general‐purpose segmentation models is sensitive to image distribution shifts, particularly variations in cell density and scale.

To support these qualitative observations with quantitative pixel‐level metrics, representative dense and sparse images were manually annotated to establish ground truth. On the densely populated image, Cellpose achieved higher segmentation accuracy (F1 = 0.7939, AP@0.5 = 0.7432) than the proposed method (F1 = 0.5270, AP@0.5 = 0.3647), confirming that general‐purpose foundation models provide superior boundary precision under dense conditions. In contrast, under sparse conditions, Cellpose performance decreased substantially (F1 = 0.2000, AP@0.5 = 0.0611), whereas the proposed method maintained more consistent performance (F1 = 0.5870, AP@0.5 = 0.4579). These results highlight the sensitivity of general‐purpose models to density‐induced distribution shifts and demonstrate the robustness of the proposed method across varying conditions. They also reveal a limitation of the proposed approach, as the edge‐based preprocessing and synthetic data training strategy result in lower boundary precision than foundation models in densely populated images.

**FIGURE 3:**
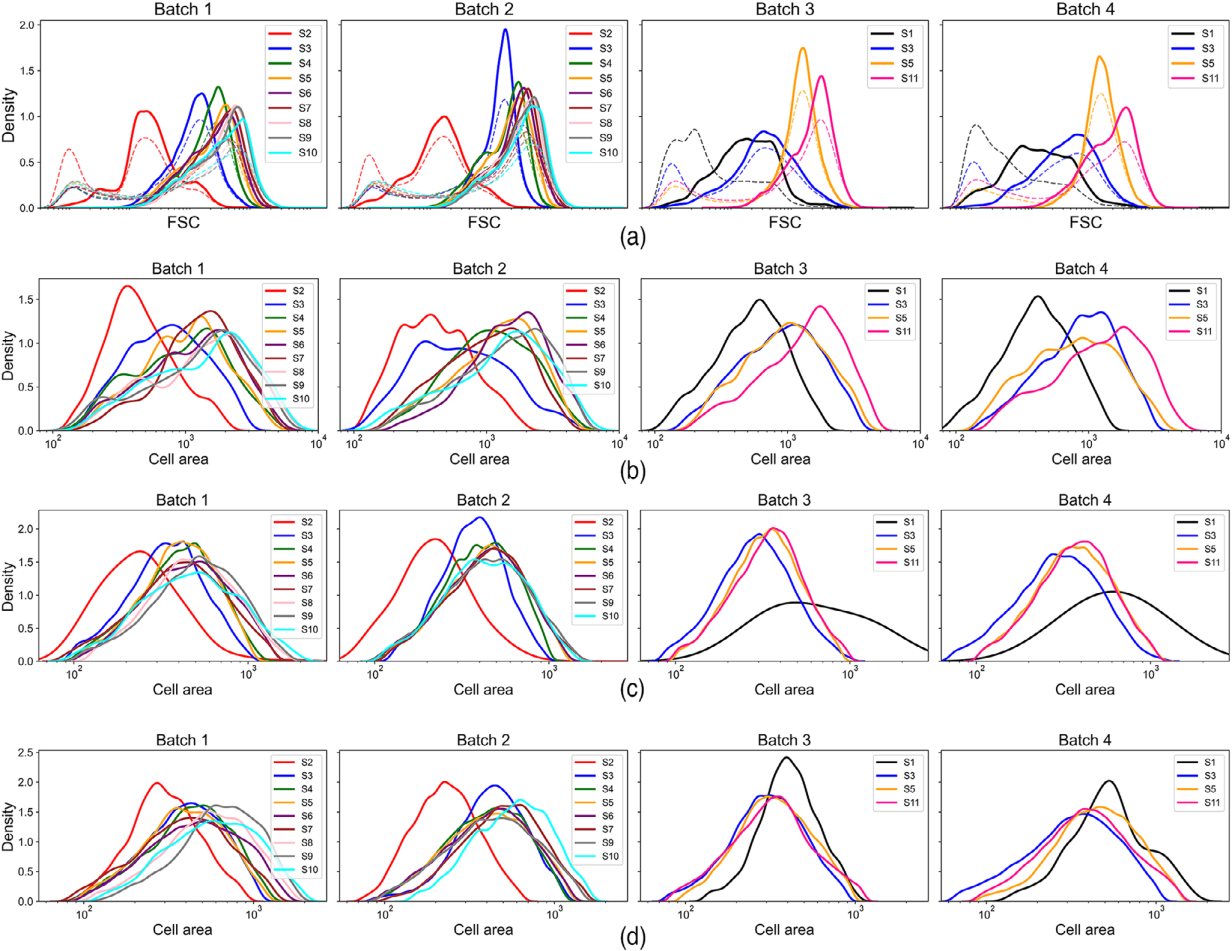
Log‐scaled Kernel density estimates (KDEs) of cell size distributions from (a) forward scatter (FSC) measurements, (b) the proposed method, (c) Cellpose, and (d) CellSAM. Each curve represents one sampling point; curves of the same color within a batch correspond to the same sample across panels. In (a), dashed curves indicate raw FSC signals before noise removal.

Figure 3 shows the log‐scaled KDE curves of the FSC‐based surrogate of the cell size distribution, together with the image‐derived cell size distributions predicted by the proposed method, Cellpose and CellSAM. In Figure 3a, the dashed curves denote the raw FSC distributions, whereas the solid curves indicate the filtered distributions. The raw FSC curves exhibit a bimodal shape, where the left peak corresponds to low‐intensity noise that is effectively removed in the post‐processed curves. As shown in Figures 3b, 3c, and 3d, all three segmentation methods generally follow the trend of the FSC‐based distribution. However, in Batch 3, the black solid lines (S1) of Cellpose and CellSAM noticeably deviate from the FSC‐based tendency, which originates from their inaccurate segmentation of sparsely populated images, as illustrated in the right panels of Figure 2b.

These discrepancies indicate that general‐purpose segmentation models can be sensitive to distribution shifts in microscopy images. In contrast, the proposed method maintains closer agreement with the FSC‐based distributions across batches, reflecting more consistent performance under varying conditions. These results suggest that task‐specific preprocessing and training are important for reliable estimation of cell size distributions in industrial bioprocess monitoring. A quantitative comparison of these trends is provided in the following section.

### 3.2 Quantitative Evaluation of Cell Size Distribution Estimation

To validate the use of cell size as a surrogate indicator for PHA accumulation, the correlation between intracellular PHA content and the log mean of FSC was first analyzed across four independent batches. The resulting correlation coefficients were high (0.9918, 0.9978, 0.9301, and 0.9865), indicating a strong positive correlation. These results confirm that cell size effectively reflects PHA accumulation under the experimental conditions, supporting its use for distribution‐based estimation.

To assess the accuracy of image‐based cell size estimation, the log mean of the predicted cell size distribution was compared with that of the FSC‐based reference at each sampling point. Pearson correlation coefficients between these two quantities were then computed across all sampling points. To further evaluate agreement at the distribution level, statistical distance metrics, including the Kolmogorov–Smirnov (K–S) statistic, Earth mover's distance (EMD), and Anderson–Darling (A–D) statistic, were computed. Prior to evaluation, each distribution was normalized under a log‐normal assumption to ensure comparability across scales. These metrics provide complementary perspectives on distribution similarity, capturing differences in cumulative behavior (K–S), and sensitivity to tail behavior (A–D). The results of these quantitative evaluations are summarized in Table 1. The FSC primarily reflects the optical cross sectional area of cells, whereas image segmentation directly measures their two‐dimensional projected areas. Although these modalities rely on different physical principles, both are highly sensitive to cell dimensions and are expected to exhibit a strong monotonic relationship. As shown in Table 1, the correlations between the mean FSC values and the mean predicted areas were consistently high, indicating an approximately linear relationship. Notably, the proposed method achieved the highest average correlation across all samples and showed higher correlations than Cellpose and CellSAM in most batches. These quantitative results support the reliability of the proposed pipeline for the microscopy images analyzed in this study.

**TABLE 1:** Quantitative evaluation of cell size distribution estimation. Pearson correlations between the log means of FSC data and predicted cell size distributions are reported alongside distribution distance metrics, including the Kolmogorov–Smirnov (K–S) statistic, Earth mover's distance (EMD), and Anderson–Darling (A–D) statistic. Arrows (↑ and ↓) indicate the direction of better performance. “Mean of batches” denotes the average of batch‐level values, while “Mean of samples” denotes the average across all sampling points. Results excluding S1 indicate that Cellpose performs well under dense conditions but degrades under sparse conditions.

Metrics	Methods	Batch 1	Batch 2	Batch 3	Batch 4	Mean of batches	Mean of samples
Corr. of log mean (↑)	Proposed method	**0.9758**	0.8940	**0.9168**	**0.8526**	**0.9098**	**0.8953**
	Cellpose	0.9140	**0.9849**	−0.7192	−0.7370	0.1107	−0.2721
	CellSAM	0.7783	0.9251	−0.5542	−0.3173	0.2080	0.5262
	Cellpose w/o S1	0.9140	0.9849	1.0000	0.9773	0.9691	0.9007
	CellSAM w/o S1	0.7783	0.9251	0.8112	0.8339	0.8371	0.8103
	Fine‐tuned Cellpose	0.9639	0.8018	−0.5995	−0.8225	0.0859	−0.1393
	Fine‐tuned Cellpose w/o S1	0.9639	0.8018	0.4299	−0.2552	0.4851	0.7190
K–S (↓)	Proposed method	0.0498	0.0610	**0.0385**	**0.0359**	**0.0463**	0.0494
	Cellpose	0.0748	0.0821	0.1177	0.1212	0.0990	0.0914
	CellSAM	**0.0484**	**0.0544**	0.0446	0.0416	0.0472	**0.0486**
EMD (↓)	Proposed method	0.0825	0.1017	**0.0771**	**0.0663**	**0.0819**	**0.0852**
	Cellpose	0.1497	0.1625	0.2340	0.2426	0.1972	0.1822
	CellSAM	**0.0803**	**0.0983**	0.0862	0.0691	0.0835	0.0852
A–D (↓)	Proposed method	**1.99**	**3.99**	**4.57**	**4.99**	**3.89**	**3.52**
	Cellpose	39.35	73.28	195.30	194.09	125.50	99.92
	CellSAM	18.94	38.92	85.11	37.25	45.06	38.85

A closer examination of the foundation models reveals their limitations under distribution shifts. As visually observed in Figures 3c and 3d, both Cellpose and CellSAM exhibited large inconsistencies for the initial sparse samples (S1) in Batch 3 and Batch 4. When these S1 samples were excluded, the average correlations for Cellpose and CellSAM increased substantially, indicating that their lower overall performance was primarily influenced by segmentation errors in sparse images. A plausible explanation is that Cellpose uses the original image directly without edge detection or binarization, thereby preserving more fine‐grained information before inference. Consequently, Cellpose can provide more discriminative predictions when image differences are subtle. In contrast, the proposed method maintains more consistent performance across diverse image distributions, whereas Cellpose achieves higher accuracy only within limited distributional ranges. These observations highlight the importance of robustness to distribution shifts for reliable estimation of cell size distributions.

Interestingly, fine‐tuning the Cellpose model using the synthetic dataset did not improve its accuracy; rather, it reduced the overall estimation performance. As detailed in Table 1, the fine‐tuned Cellpose exhibited lower correlations. Even when sparse images (S1) were excluded, the mean correlation of the fine‐tuned Cellpose remained lower than that of the pre‐trained Cellpose. This observation suggests a mismatch between the synthetic training data and the feature extraction mechanisms of gradient‐dependent models. Since the synthetic data generated by the proposed pipeline is based on edge detection and binarization to emulate realistic dense structures, it lacks the raw spatial gradients and fine‐grained intensity variations present in original microscope images. Training a gradient‐dependent model such as Cellpose on such data may therefore degrade its performance. These results indicate that the effectiveness of synthetic data is closely related to its compatibility with the underlying model architecture, and the proposed pipeline is better aligned with architectures such as Mask R‐CNN.

**FIGURE 4:**
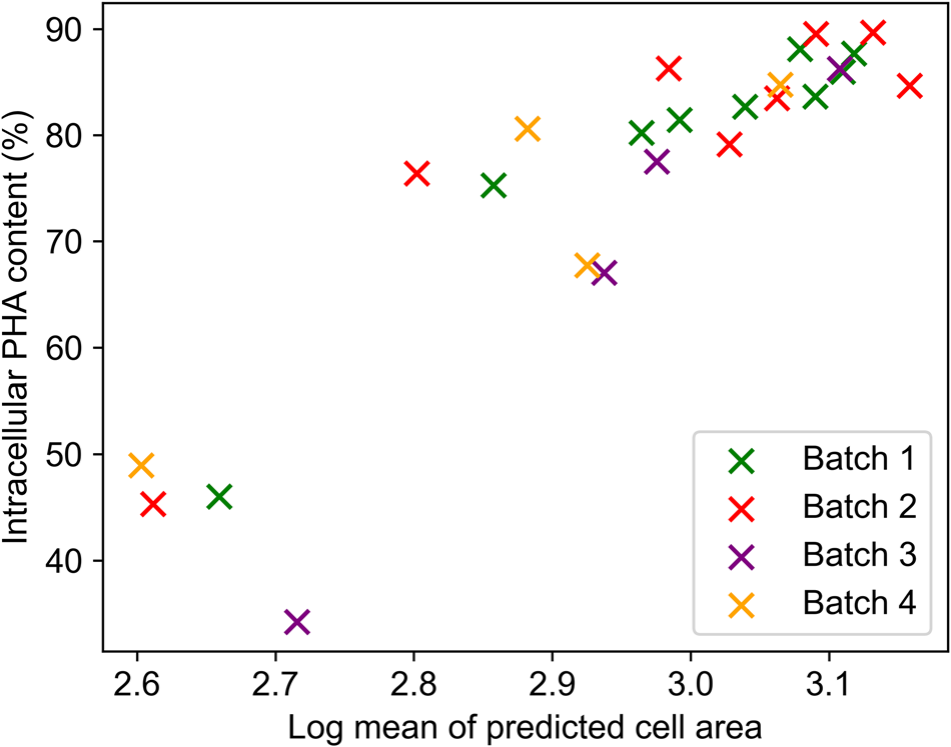
Direct relationship between the proposed pipeline‐derived cell‐size metric and GC‐measured intracellular PHA content. The *x*‐axis represents the log mean of the predicted cell‐area distribution obtained from the proposed pipeline, and the *y*‐axis represents intracellular PHA content measured by GC‐FID. Each point corresponds to one sampling point with paired microscopy and GC measurements, with colors indicating different batches. The pooled Pearson correlation coefficient across all sampling points was 0.8974, and the batch‐wise coefficients were 0.9510, 0.9018, 0.9838, and 0.9096 for Batch 1, Batch 2, Batch 3, and Batch 4, respectively.

To further close the validation loop between the image‐derived cell‐size metric and the biochemical PHA readout, the log mean of the cell‐area distribution predicted by the proposed model was directly compared with GC‐measured intracellular PHA content across the four evaluation batches (Figure 4). All sampling points with paired microscopy and GC measurements were pooled in a single scatter plot, with batches color‐coded. The Pearson correlation coefficient across all sampling points was 0.8974, indicating a strong positive relationship between the proposed model‐predicted log mean cell area and GC‐measured intracellular PHA content. Because the evaluation batches involved different batch‐specific experimental and imaging conditions, batch‐wise correlations were also calculated to examine whether this relationship was consistently maintained within individual batches. The Pearson correlation coefficients were 0.9510, 0.9018, 0.9838, and 0.9096 for Batch 1, Batch 2, Batch 3, and Batch 4, respectively. These results provide direct evidence that the proposed vision pipeline captures a biologically relevant image‐derived size signal associated with intracellular PHA accumulation. Furthermore, they establish a direct link between the model‐predicted cell‐size metric and the biochemical PHA measurement, complementing the previously reported relationship among predicted cell size, FSC, and PHA content.

### 3.3 Sensitivity Analysis of Empirical Parameters

To evaluate the dependence of the proposed pipeline on hand‐crafted parameters, sensitivity analyses were conducted for both the synthetic data generation stage and the cell size distribution prediction stage.

**FIGURE 5:**
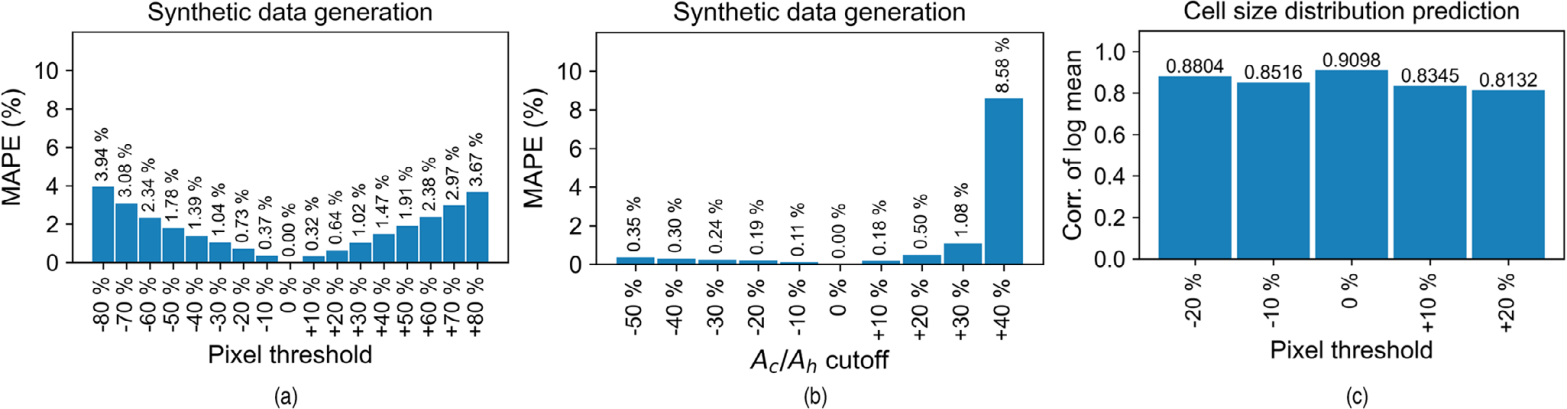
Sensitivity analysis of the empirical parameters in the proposed pipeline. Mean absolute percentage error (MAPE) of the log mean cell area when varying: (a) the pixel threshold and (b) the *A_c_*/*A_h_* cutoff during synthetic data generation. (c) Pearson correlation of log means between FSC data and the predicted cell sizes when varying the pixel threshold during the Mask R‐CNN inference.

For synthetic data generation, parameter sensitivity was assessed using the MAPE of the log mean cell area across 2913 sparse images. Varying the pixel binarization threshold by −80% to +80% from its baseline value (100) resulted in deviations below 4% (Figure 5a). Similarly, the convexity‐based cutoff (*A_c_*/*A_h_*, baseline 0.70) exhibited minimal sensitivity across a −50% to +30% variation range, with MAPE remaining below 1% (Figure 5b). A noticeable deviation (8.58%) was observed only at an extreme +40% variation (cutoff = 0.98). As this cutoff value approaches 1, only strictly convex objects are retained, leading to exclusion of naturally curved cells and a systematic bias in the resulting size distribution.

For the cell size distribution prediction stage, robustness was evaluated by varying the pixel threshold during inference and comparing the predicted distributions with FSC‐based reference data using Pearson correlation. Across a ±20% variation range, the model maintained stable performance (correlation > 0.81) (Figure 5c). Although the highest performance was achieved at the baseline threshold used during training, the results indicate that the model generalizes well to moderate deviations in this parameter.

These results suggest that the proposed pipeline is not highly sensitive to moderate variations in key empirical parameters. Parameters such as size‐based filtering thresholds can be adjusted based on expected cell size and imaging magnification without requiring extensive tuning.

## 4 Discussion

The widespread industrial adoption of PHA bioprocesses is heavily dependent on the development of reliable, real time online monitoring systems. While monitoring cell size distribution offers a strong surrogate for intracellular PHA content, implementing deep learning‐based segmentation in industrial settings is severely bottlenecked by the difficulty of manually annotating densely populated microscopy images. This challenge is addressed through an automated, annotation‐free synthetic data generation pipeline. By extracting precise single‐cell crops from diluted samples and compositing them onto heterogeneous backgrounds, realistic dense‐image conditions are reconstructed without manual annotation.

Importantly, the dataset utilized in this study incorporates multiple independent biological batches cultivated across different bioreactor scales, including industrial‐scale systems and one laboratory‐scale batch used for synthetic data generation. As fermentation conditions varied across batches, the dataset captures realistic variability in imaging conditions, such as changes in cell density and background turbidity, which commonly arise during biomanufacturing. In this context, robustness refers to the ability of the model to maintain consistent performance under such process‐induced image distribution shifts. A key observation is the difference in behavior between task‐specific models and general‐purpose foundation models. While foundation models such as Cellpose and CellSAM possess strong zero‐shot capabilities, the results indicate their sensitivity to variations in cell density, often leading to over‐segmentation in sparse conditions. In contrast, the proposed Mask R‐CNN model, trained on the synthetic dataset, maintains more consistent performance across both dense and sparse conditions, and more closely follows FSC‐based distribution trends. These results suggest that, for industrial applications with dynamically varying imaging conditions, task‐specific data representations remain important for reliable performance.

However, foundation models also retain specific advantages. For instance, Cellpose occasionally yielded higher correlation metrics on specific dense sample sets when sparse outliers were excluded, likely because its end‐to‐end processing preserves fine‐grained spatial gradients that are reduced during edge enhancement and binarization. This observation suggests a trade‐off between the robustness provided by the preprocessing‐based synthetic pipeline and the localized precision of gradient‐based models.

A promising direction for future research is the exploration of hybrid approaches that combine the density robustness of the proposed pipeline with the adaptability of foundation models. For example, employing foundation models as a secondary refinement step on individual cell crops may further improve segmentation boundaries while preserving robustness to image distribution shifts.

## Author Contributions

**Han Bit Kim**: conceptualization, methodology, visualization, investigation, writing – original draft, review and editing. **Chaeeun Lee**: investigation, writing – original draft, review and editing. **Naeun Lee**: resources, data curation. **Hyeongseok Han**: resources, data curation. **Chanhun Park**: resources, data curation. **Moo Sun Hong**: conceptualization, methodology, supervision, funding acquisition, writing – review and editing.

## Conflicts of Interest

The authors declare no conflicts of interest.

## Data Availability

The data used in this study are not publicly available due to confidentiality and proprietary restrictions.

## Endnotes

1
https://github.com/xavysp/DexiNed

2
https://github.com/facebookresearch/detectron2
